# Importance of International Networking and Comparative Research in Screening to Meet the Global Challenge of Cancer Control

**DOI:** 10.1200/JGO.19.00388

**Published:** 2020-01-31

**Authors:** Mireille Broeders, K. Miriam Elfström

**Affiliations:** ^1^ Radboud University Medical Center, Radboud Institute for Health Sciences, Nijmegen, the Netherlands; ^2^ Dutch Expert Centre for Screening, Nijmegen, the Netherlands; ^3^ Department of Laboratory Medicine, Karolinska Institutet, Stockholm, Sweden; ^4^ Regional Cancer Center of Stockholm-Gotland, Stockholm, Sweden

Cancer prevention and early detection are central to worldwide efforts to control the burden of cancer in our populations. The adoption and implementation of best practices in cancer screening, and timely research to address new questions, holds great potential to reduce morbidity and mortality from cancer around the globe. Sharing knowledge and experiences across different cancer screening programs and different settings can accelerate more effective screening implementation and evaluation and identify research opportunities to advance cancer prevention and control. In October 2019, two publications in this journal described the value of international research networks and, more specifically, the International Cancer Screening Network (ICSN).^1,2^

The ICSN began with an initiative of Prof Sam Shapiro to create a common database for the evaluation of breast cancer screening programs.^3^ This seemingly straightforward task was, in fact, a very ambitious one. Comparing outcomes of programs without country-specific contexts, such as differences in health care systems, organization of screening activities, screening policies, and follow-up results, leads to comparisons that may mischaracterize program effectiveness and obscure strengths and potential development opportunities. At the same time, the process of trying to understand these differences and how they might affect outcomes of screening is extremely helpful to establish a standardized approach in comparisons of screening programs.

With support from the US National Cancer Institute (NCI), the ICSN started as the International Breast Cancer Screening Network (IBSN). The inaugural meeting of the IBSN was held in 1988 with representatives from 11 countries. By 2005, IBSN membership had grown to 27 countries. In May 2006, the scope of the network was expanded to include additional cancer sites, spurred by the fact that many IBSN members were already becoming involved in cervical and colorectal cancer screening programs. Consequently, the network was renamed to the ICSN.^4^ The ICSN has become even more expansive in recent years, including sessions at its biennial meetings on prostate cancer screening, lung cancer screening, and oral cancer screening. Inclusion of the latter cancer site also reflects the growing aspiration of the ICSN to reach out and include screening professionals from low- and middle-income countries (LMICs). As LMICs are increasingly affected by growing numbers of patients with cancer, the opportunity to learn from experiences of the countries with more established screening programs may accelerate effective implementation and evaluation of their cancer control programs. At the same time, learning from the experiences of LMICs may also encourage re-evaluation of cancer screening programs in high-income settings.

Broadly, results from the 2018 survey by Puricelli Perin et al^2^ of ICSN participants showed that the distribution of years of experience in the field of cancer screening, work activities, and workplace affiliations differed between HICs and LMICs. HIC respondents had more years of experience in the cancer screening field, were more likely to be engaged with research, and worked primarily in academic settings. In contrast, LMIC respondents spent more time directly involved with various aspects of implementation and management of screening programs and worked primarily in government agencies. Differences were also observed with regard to cancer site of interest. HIC respondents were interested in colorectal, lung, and prostate cancers, whereas LMIC respondents were more interested in cervical cancer. As the authors note, these differences are consistent with the distribution of cancer burden between HICs and LMICs and the emergence of screening programs in LMICs in recent years. Respondents from both HICs and LMICs reported that the ICSN, through its biennial meetings and scientific working groups, fosters collaboration and facilitates networking and knowledge sharing. Notably, LMIC respondents reported that the ICSN particularly facilitated the receipt of technical assistance for screening implementation in their settings.^2^

These survey results illuminate the many contributions of the ICSN. However, as the network has grown and the field of cancer screening has enlarged to include more cancer sites, meeting the needs and interests of members across programs and settings has become more challenging, particularly for setting biennial meeting agendas. What began as a small, collaborative group of breast cancer screening researchers has expanded to encompass a much larger and more diverse group of dedicated professionals representing different aspects of research, implementation, monitoring, and evaluation. The spirit of knowledge sharing and networking remains, as evidenced by the survey results, but we believe that the ICSN could be further strengthened through increased focus on the efforts of the scientific working groups at future meetings and support of their continued work between meetings. Historically, ICSN working group initiatives with support from the NCI have resulted in the publication of 21 papers comparing outcomes of cancer screening programs across participating countries and have advanced other collaborative research efforts. Future working group efforts could support early-career researchers interested in screening by providing opportunities to actively engage in collaborative projects, define relevant research topics, and further facilitate knowledge sharing and networking across settings.

The mission of the ICSN is to reduce the burden of cancer by promoting dialogue and collaborative cancer screening research and evaluation regarding the effectiveness of context-specific evidence-based cancer screening programs, including promotion, recruitment and follow-up, among other relevant topics. Recent evidence suggests that cancer will surpass cardiovascular disease as the leading cause of death in many HICs and some middle-income countries.^5^ By providing an inclusive forum for dedicated screening professionals to collaborate in cancer screening research and evaluation, engage in knowledge sharing, and mentor the next generation of screening professionals, the ICSN is uniquely positioned to play an important role in global cancer control.
